# CDK4/6 inhibitors have potent activity in combination with pathway selective therapeutic agents in models of pancreatic cancer

**DOI:** 10.18632/oncotarget.2270

**Published:** 2014-07-26

**Authors:** Jorge Franco, Agnieszka K. Witkiewicz, Erik S. Knudsen

**Affiliations:** ^1^ Department of Pathology, UT Southwestern, Dallas TX; ^2^ Simmons Cancer Center, UT Southwestern, Dallas TX

**Keywords:** RB, CDK4/6, palbocicllb, pancreatic cancer, e2f

## Abstract

Pancreatic ductal adenocarcinoma (PDA) has a poor prognosis, in part, due to the therapy-recalcitrant nature of the disease. Loss of the CDK4/6 inhibitor CDKN2A is a signature genetic event in PDA. Therefore, PDA may be amenable to treatment with pharmaceutical CDK4/6 inhibitors. Surprisingly, response to CDK4/6 inhibition was highly variable in PDA models, and associated with differential suppression of gene expression. Mitotic genes were repressed and FOXM1 was uniformly attenuated; however, genes involved in DNA replication were uniquely suppressed in sensitive models. Aberrant induction of Cyclin E1 was associated with resistance, and knockdown demonstrated synergistic suppression of the cell cycle with CDK4/6 inhibition. Combination therapies are likely required for the effective treatment of disease, and drug screening demonstrated additive/antagonistic interactions with CDK4/6 inhibitors. Agents dependent on mitotic progression (taxanes/PLK1 inhibitors) were antagonized by CDK4/6 inhibition, while the response to 5-FU and gemcitabine exhibited drug specific interactions. PI3K/MTOR and MEK inhibitors potently cooperated with CDK4/6 inhibition. These agents were synergistic with CDK4/6 inhibition, blocked the aberrant upregulation of Cyclin E1, and yielded potent inhibition of tumor cell growth. Together, these data identify novel mechanisms of resistance to CDK4/6 inhibitions and provide a roadmap for combination therapies in the treatment of PDA.

## INTRODUCTION

Pancreatic ductal adenocarcinoma (PDA) has a terrible prognosis with a 5-year survival of approximately 6% [1-3]. The approved systemic therapies have a relatively modest impact on survival, and PDA is considered a therapy recalcitrant disease [1, 2, 4]. Interestingly, the treatment of PDA has remained largely dependent on the use of systemic chemotherapy regimens, and there are basically no targeted approaches to treatment that exploit the underlying genetic features of pancreatic cancer. PDA is largely driven by oncogenic events (e.g. KRAS), which historically are considered “non-actionable” from a therapeutic perspective. However, PDA exhibits a range of genetic alterations some of which could be amenable to targeted therapy. One of these alterations is the genetic loss or epigenetic silencing of the CDKN2A tumor suppressor [5-8].

The CDKN2A gene encodes the p16ink4a protein that is a potent inhibitor of Cyclin Dependent Kinases 4 and 6 (CDK4/6). Physiologically, p16ink4a represents a key barrier to oncogenic transformation, as it is induced by oncogenic stress and leads to senescence in multiple disease relevant settings [9]. In the context of PDA, it has been hypothesized that p16ink4a loss is selected for to enable the progression of KRAS mutated cells [10-13]. Correspondingly, it has been shown that the over-expression of p16ink4a is dominant to the effects of KRAS in cell culture models and is capable of re-establishing a senescence-like arrest in established cancer models [14-17]. The only known functional target of p16ink4a are the kinases CDK4 and CDK6, and a plethora of data support this concept [9, 18-23]. For example, p16ink4a-mediated arrest is selectively bypassed by CDK4 mutations that disrupt the association with the inhibitor [24, 25]. Similarly, loss of RB, which is the down stream target for CDK4/6 bypasses the growth inhibitory activity of p16ink4a [9, 26]. Furthermore, analysis of mutual-exclusivity in cancer demonstrates that there is a pronounced reciprocal relationship between the loss of p16ink4a, deregulation of CDK4/6, and loss of RB [25, 27, 28]. Thus these events describe a single pathway, wherein the predominant event occurring in PDA is loss of p16ink4a, and suggest that restoring its biological function could represent a key means to limit the growth of KRAS driven cancers.

While multiple CDK-inhibitory agents have been evaluated in clinical trials, only recently have highly specific CDK4/6 inhibitory drugs been developed [29, 30]. Consistent with the function of p16ink4a, they induce a highly potent G1-arrest that is dependent on the suppression of CDK4/6 and the presence of RB tumor suppressor (RB) [31-33]. RB is a critical downstream effector of CDK4/6 and regulates the expression of a host of target genes through interactions with E2F and other transcription factor complexes [34]. These targets include CDK/Cyclin subunits (e.g. Cyclin E and Cyclin A), DNA replication factors (e.g. MCM7 and PCNA), genes involved in dNTP metabolism (e.g. thymidylate synthase and ribonucleotide reductase), and mitotic progression (e.g. PLK1 and CDC20). In preclinical models, activation of RB via CDK4/6 inhibition can induce senescent-like arrest [29, 30, 35-39]. An important parallel effector of CDK4/6 is FOXM1, which is stabilized by direct CDK4/6 mediated phosphorylation and stimulates expression of cell cycle regulated genes [40, 41]. Recently, clinical studies have demonstrated that CDK4/6 inhibitors can have potent single agent activity in select tumor models ostensibly addicted to kinase activity, such as liposarcoma and mantle cell lymphoma [42-45]. Additionally, in breast cancer CDK4/6 inhibitors have demonstrated highly significant activity in combination with endocrine agents [46-50]. However, it is also clear that there are features of tumor behavior that we do not fully understand, as specific diseases which frequently lose p16ink4a had minimal response to CDK4/6 inhibitors in the clinic [44, 45].

Here we find that CDK4/6 inhibition can have a potent impact on PDA models. While some models exhibit a durable response, acquired/intrinsic resistance can bypass the action of CDK4/6 inhibition in the majority of models analyzed via a novel mechanism involving induction of Cyclin E1. Drug screening reveals a complex and mechanism specific impact of CDK4/6 inhibitors on drug-sensitivity. However, specific combination therapies clearly expand the therapeutic potency of CDK4/6 inhibition for the treatment of PDA.

## RESULTS

### CDKN2A-deficient PDA models exhibit differential response to CDK4/6 inhibition

**Figure 1:**
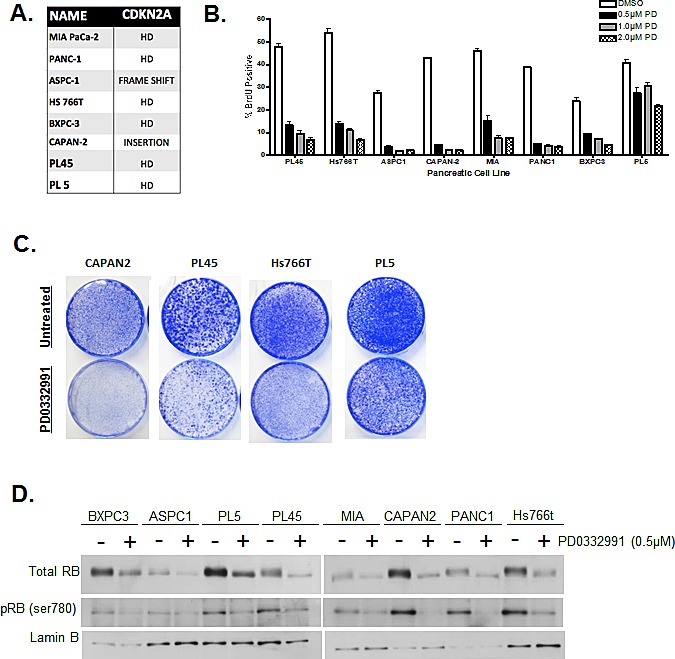
PDA cell lines display differential responses to CDK4/6 inhibition (A) Eight PDA cell lines were employed with documented deleterious mutations in the CDKN2A tumor suppressor. (B) Cells were treated with indicated concentrations of PD-0332991 (0.5, 1, and 2 μM) for 24 hours. Cell cycle progression was quantified by BrdU incorporation as determined by flow cytometry. Data shown is from three independent experiments. (C) CDK4/6 effects on total RB and phospho-serine 780 were evaluated by immunoblotting. (D) Long-term responses to PD-0332991 in select PDA cell lines were assessed using crystal violet staining after 10 days of treatment with drug replenishment every three days.

Eight pancreatic cancer cell lines were screened for their proliferative response to CDK4/6 inhibition. In addition to multiple PDA signature mutations (e.g. KRAS, SMAD4, and TP53) these cell lines carried a non-functional CDKN2A gene (Fig. 1A). In order to test sensitivity to CDK4/6 inhibitors, cells were treated with increasing concentrations of PD-0332991, and proliferation was measured by 5-bromo-2-deoxyuridine (BrdU) incorporation (Fig. 1B). Results showed that some model experienced a complete suppression of proliferation (e.g. CAPAN2), while others were modestly or mostly resistant to CDK4/6 inhibitor (e.g. HS766T and PL5). Recognizing that PDA cell lines display differential proliferative responses to acute treatment, long-term treatment responses were also monitored in order to evaluate if the responses are maintained over time (Fig 1C). CAPAN2 cells being the most sensitive to the acute treatment, also exhibited significant suppression of proliferation with long-term treatment. In contrast, cell proliferation occurred in PL45, ASPC1 and PL5, albeit at a reduced extent relative to control. These results indicate that there is differential proliferative response to CDK4/6 inhibition within CDKN2A deficient PDA cell models, and suggests that there are additional determinants of therapeutic sensitivity. A possible explanation of these results is that in resistant models the phosphorylation of RB is CDK4/6 independent. In analysis of RB phosphorylation by mobility shift and with phospho-specific antibodies, CDK4/6 inhibition did suppress the phosphorylation of RB across all models interrogated (Fig 1D). These results were confirmed by reverse phase protein array (RPPA) analysis, wherein RB dephosphorylation was the only consistently observed change amongst 53 phosphoproteins analyzed (not shown). Thus, from conventional assessment of the RB-pathway, CDK4/6 inhibitors should be actionable.

### PDA models exhibit distinct suppression of E2F gene expression with CDK4/6 inhibition

**Figure 2:**
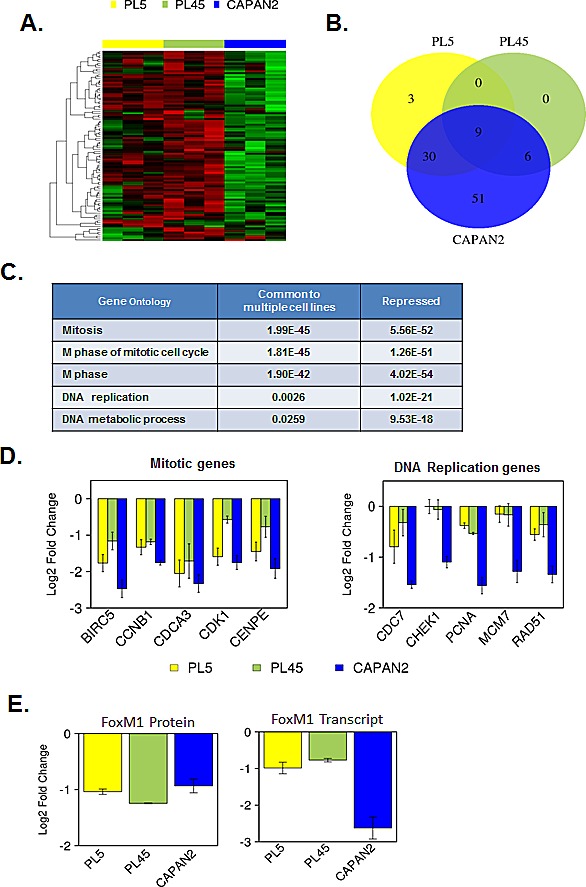
Gene expression profiling of RB/E2F target genes reveals differential transcriptional repression (A) Heatmap analysis of transcripts that are potently repressed by PD-0332991 in at least one cell model. (B) Venn diagrams demonstrating the overlap of genes that are repressed by at least two-fold (p<0.05) in each cell line. (C) Gene ontology analysis of genes repressed in multiple models vs. those repressed in CAPAN2. (D) Representative gene expression of genes involved in mitotic progression (left) and DNA replication replication (right).

To interrogate the downstream signaling from CDK4/6 inhibition, we analyzed the expression of RB/E2F target genes using a high-density RBTARGET Affymetrix array (probes listed in supplemental tables). Using a 2-fold cutoff and p<0.05 we found that there were many RB/E2F targets genes that were repressed potently with CDK4/6 inhibition (Fig. 2A). Specifically, a large number of genes were repressed in CAPAN2 cells that exhibit a durable response to CDK4/6 inhibition. In contrast, the PL45 and PL5 cell lines exhibited modest suppression of such genes (Fig. 2B). Interestingly, by gene ontology analyses these groups of genes segregated along biological processes (Fig. 2C). There was potent suppression of mitotic genes across cell models with CDK4/6 inhibition; however, the suppression of DNA replication proteins was largely restricted to CAPAN2. Bar-graphs demonstrating the repression of representative genes are shown (Fig. 2D). One of the key determinants of mitotic gene expression is FOXM1 [41]. FOXM1 is both a transcriptional target of RB/E2F, and a direct target for protein stability by CDK4/6 [40]. By gene expression profiling FOXM1 repression was largely restricted to CAPAN2 cells; however, by RPPA analysis FOXM1 protein levels were reduced uniformly (Fig. 2E). These data suggest that there is a fundamental difference in the mechanisms underlying the repression of different classes of cell cycle genes, but that suppression of genes involved in DNA replication was particularly associated with durable response to CDK4/6 inhibition.

### Intrinsic aberrant regulation of Cyclin E is a key determinant of resistance to CDK4/6 inhibition

**Figure 3:**
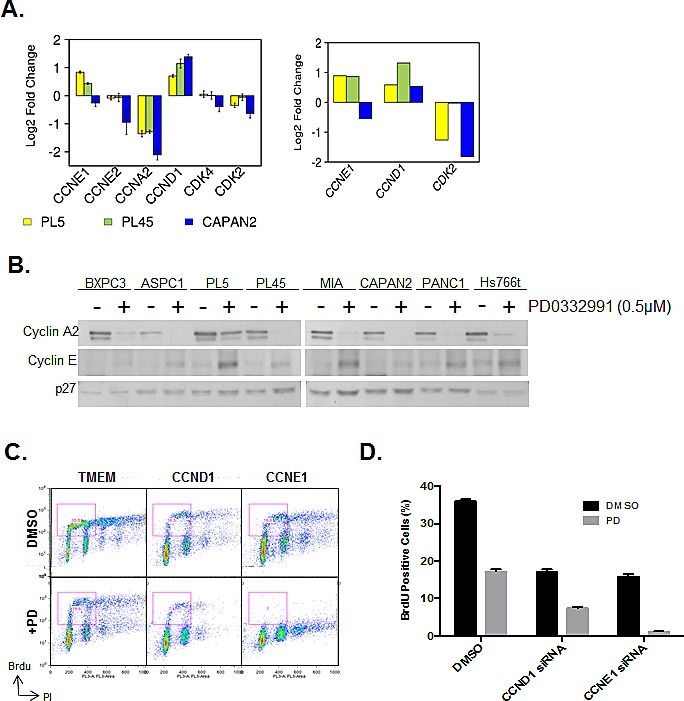
CDK4/6 inhibition induces aberrant expression of CCNE1 and CCND1 (A) Analysis of gene expression related to control over G1/S regulated CDK activity is shown from two different array platforms. (B) CCNE1 expression was evaluated by immunoblotting. (C) Cell cycle progression of PL5 cells was determined following knockdown of CCND1 or CCNE1 by RNAi in the presence or absence of 1μM PD-0332991. (D) Quantification of BrdU incorporation from three independent experiments. Data is expressed as percentage of BrdU positive cells, and a significant difference (p<0.01) was observed between CCND1 and CCNE1 knockdown concurrent with PD-0332991 treatment versus knockdown alone in both PL-45 and PL-5.

In an attempt to decipher the mechanism underlying the specific resistance to CDK4/6 inhibition, we interrogated the expression of critical CDK and Cyclins that are important for progression through G1/S. These data demonstrated that certain genes are repressed irrespectively (e.g. Cyclin A2) (Fig. 3A). However, there was a consistent upregulation of Cyclin D1 in all models (Fig 3A). Additionally in PL45 and PL5 cells that are relatively resistant to CDK4/6 inhibition there was a reproducible upregulation of Cyclin E1 transcript (Fig 3A). The induction of Cyclin D1 has been previously described in other models [32, 33]; however, the induction of Cyclin E1 is a unique observation in the setting of PDA. Therefore, the levels of Cyclin E1 were determined by immunoblotting across all cell lines (Fig. 3B). These data revealed that cyclin E1 induction was particularly apparent in multiple cell lines including PL5, MIAPACA2, PANC1 and HS766T. To determine the functional relevance of cyclin upregulation in PDA models exposed to PD-0332991, RNAi was used to knockdown Cyclin D1 and Cyclin E1. Individually, both Cyclin D1 and Cyclin E1 knockdown inhibited cell cycle progression as determined by BrdU incorporation (Fig. 3C/D). In the case of Cyclin D1 depletion there was an additive effect on cell cycle progression in combination with PD-0332991. In contrast, Cyclin E1 deletion prevented virtually all BrdU incorporation in combination with PD-0332991. These data indicate that Cyclin E1 upregulation contributes to the observed resistance of PDA models to CDK4/6 inhibition, and suggests a unique feature of signaling that compromises the sensitivity to CDK4/6 inhibition in PDA models.

### CDK4/6 inhibition results in both antagonistic and additive responses with other anti-cancer therapies

**Figure 4:**
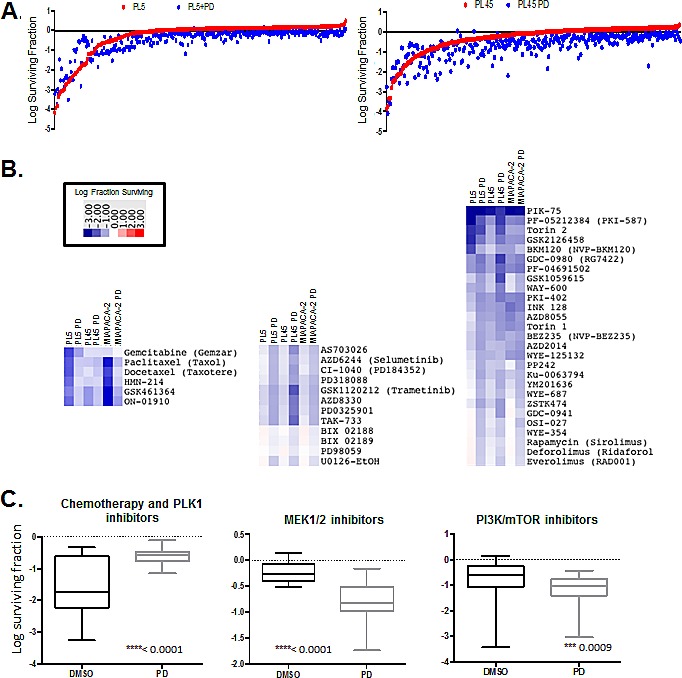
Drug screen for interaction with CDK4/6 inhibitor (A) Scatter plots diagrams showing overall drug responses to drug screen by PL5, PL45 and MIAPACA2 in the presence or absence of 1μM PD-0332991. (B) Heatmaps displaying relative response to chemotherapy/Plk1 inhibitors (left), MEK inhibitors (middle) and PI3K/MTOR inhibitors (right) (C) Log surviving fraction chemotherapy/Plk1 inhibitors (left), MEK inhibitors (middle) and PI3K/MTOR inhibitors (right) for all cell lines. Average, 95% confidence interval and p-value are shown.

It is widely recognized that combination approaches to therapy will be required for the success of targeted agents in the treatment of pancreatic cancer. Given the modest response to CDK4/6 inhibitors we interrogated the impact of PD-0332991 on 304 anti-cancer compounds (supplemental tables). Drug screening was performed in PL45, PL5 and MIAPACA2 PDA models, which are relatively resistant to PD-0332991. Cells were treated singly with the drug library or with concurrent PD-0332991. Cell survival was determined by CTG analysis and representative data is depicted in the scatter plots (Fig. 4A). To globally evaluate the response of select classes of drugs, heatmaps were generated plotting the log fraction of surviving cells to define the relative response (Fig. 4B). Antagonistic responses are drug combinations where CDK4/6 inhibitor protected cells from the other agent. We observed robust antagonism of specific chemotherapeutic drugs, PLK1 inhibitors, and other drugs specifically dependent on ongoing cell cycle progression for their mechanism of action (Fig. 4C, left panel). In contrast, additive effects were observed for MEK across a relatively broad range of compounds (Fig. 4B and C, middle). Similarly PI3K (e.g. GDC0941), MTOR (e.g. AZD0855), and dual PI3K/MTOR (e.g. GDC0980) inhibitors exhibited cooperative inhibition with PD-0332991 (Fig. 4B and C, right). In all cases the overall impact of CDK4/6 inhibition was statistically significant across the drug class selected.

### Drug context specific effects of CDK4/6 inhibition on chemotherapies

**Figure 5:**
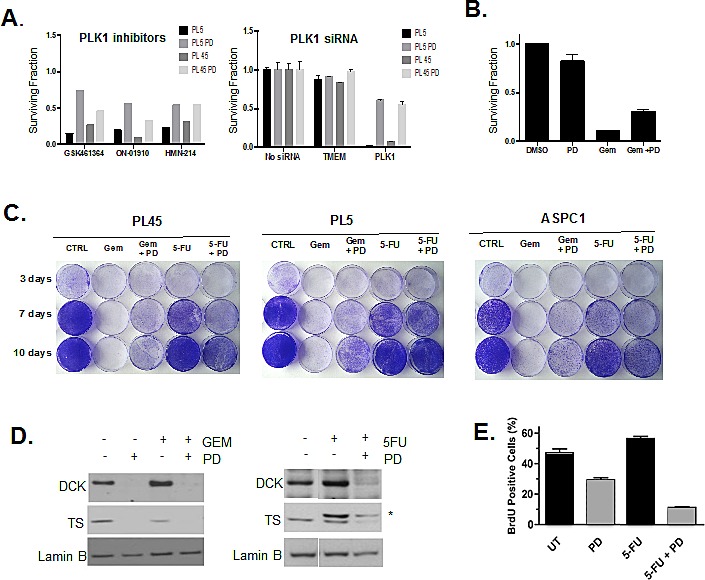
Drug specific effect of CDK4/6 inhibition on chemotherapy (A) PL5 cells were either treated with PLK1 inhibitors or transfected with the indicated RNAi in the presence or absence of 1 μM PD-0332991. At 72 hours post-treatment or transfection cell viability was measured by Cell-titer glow. Results are expressed as the surviving fraction, and a significant difference of p<0.005 was observed between co-treated with PD-0332991 compared to PLK1 inhibited alone. (B) PL-5 cells were treated with 5 μM gemcitabine as a single agent or in combination with PD-0332991 for 72 hours and viability assessed by Cell titer Glo. Results are expressed as the surviving fraction, and a significant difference of p<0.005 was observed between co-treated with gemcitabine and PD-0332991 compared to gemcitabine treatment alone. (C) Long-Term responses to single agent or co-treatments with Gemcitabine (1μM) and 5-FU (25 μM) in the presence or absence of 1 μM PD-0332991 was evaluated in PL-45, PL5 and ASPC1 cells by crystal violet. (D) Effects of CDK4/6 inhibition concurrent with either Gemcitabine or 5-FU on the DCK and TS protein was detected by immunoblotting. **Indicates the mobility of 5FU-TS adduct. (E) PL5 Cells were treated as indicated with PD-0332991 (1μM) or 5-FU (25 μM).

The drug screening indicated that CDK4/6 inhibition has the potential to antagonize the effect of various types of chemotherapies. We speculated that the cytostatic effect of CDK4/6 inhibition may represent the principle mechanism leading to a decreased efficacy of drugs targeting the mitotic machinery. As shown, PLK1 inhibitors were antagonized by co-treatment with PD-0332991 (Fig. 5A). Since such drugs can have off-target effects, PLK1, which is an essential kinase during mitosis, was depleted (Fig. 5A). In absence of CDK4/6 inhibitors, PLK inhibition led to significant reduction in viability, but in the presence of CDK4/6 inhibitor, this effect was significantly diminished. This finding suggested that even the modest cell cycle inhibition observed in PL5 and PL45 models will still impinge on the cytotoxic effects of anti-mitotic agents, such as docetaxel and paclitaxel that are present in the drug screen.

In addition to anti-mitotic drugs, PD-0332991 treatment significantly antagonized Gemcitabine activity in drug screening. Since the anti-metabolites Gemcitabine and 5-FU are both used in the treatment of PDA, we specifically evaluated response to these drugs. Initially, we simply recapitulated the antagonism of Gemcitabine-mediated toxicity (Fig. 5B). These short-term effects were re-affirmed in the analysis of overall cell survival by crystal violet analysis (Fig. 5C). In this setting, Gemcitabine effectively killed the PDA cells; however, in the presence of PD-0332991 a fraction of cells survived. These findings contrasted with the observation for 5-FU, which although having limited effect, was not antagonized by CDK4/6 inhibition and in some lines there was evidence of cooperative effects (Fig. 5C). A likely basis for this differential response relates to the role of E2F target genes deoxycytidine kinase (DCK) and thymidylate synthase (TS). DCK is required to activate gemcitabine and cells exposed to PD-0332991 have reduced level of this protein (Fig. 5D). In contrast TS is the target for 5FU-mediated inhibition and reduced levels of TS are associated with increased drug sensitivity. In the PDA models, treatment with PD-0332991 suppressed TS levels (Fig. 5D). Treatment with 5FU leads to a covalent bond with TS, and thus stoichiometric inhibition of TS is ostensibly easier to achieve. Consistent with this hypothesis, we observed additive effects between PD-0332991 and 5FU and the suppression of BrdU incorporation (Fig. 5E). Together, these findings indicate that while combination treatments of CDK4/6 inhibitor and chemotherapy can be antagonistic, there are specific conditions where additive responses can be identified based on mechanism of action.

### Concurrent inhibition of CDK4/6 either with PI3K/mTOR or MEK inhibitors display cooperation in suppression of PDA proliferation

**Figure 6:**
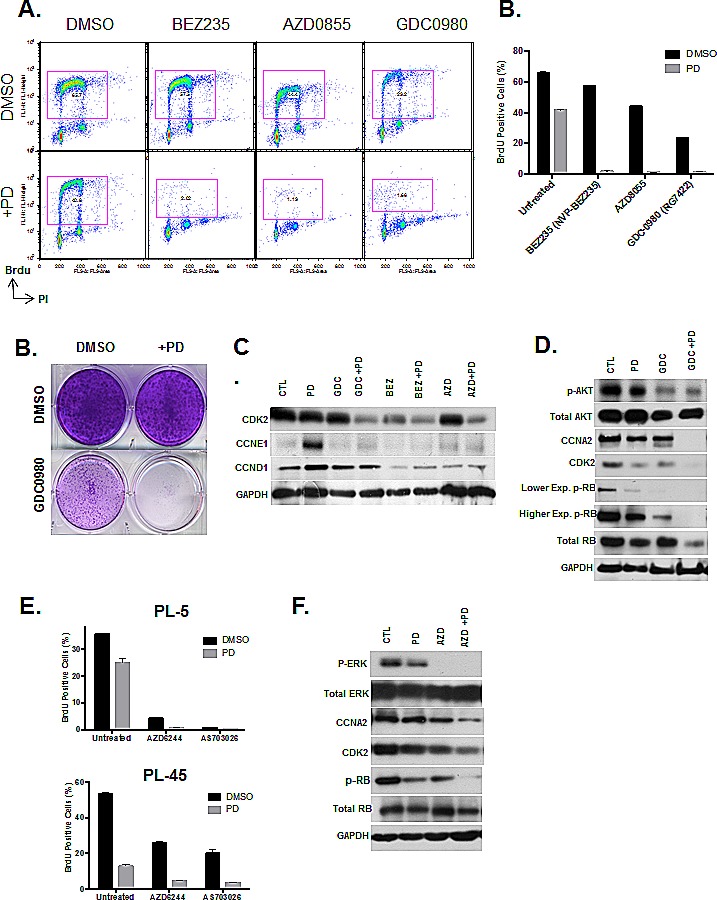
PI3K/MTOR and MEK inhibitors synergize with CDK4/6 inhibition (A) PDA model PL5 was treated with 1μM PI3K/MTOR inhibitors (BEZ235, AZD0855 and GDC0980) as a single agent or co-treated with 1μM PD-0332991. Cell cycle progression was determined by flow cytometry. (B) Quantification of BrdU incorporation from three independent experiments. Results are expressed as percentage of BrdU positive cells, and a significant difference of p<0.001 was observed between co-treated versus PI3K inhibitor treatment alone. (C) Treatment with GDC0980 as a single agent and in combination with PD-0332991 was evaluated by crystal violet. (C) Effect of treatment with the PI3K/MTOR inhibitors alone and in the presence of PD-0332991 on the expression of the indicated proteins was determined by immunoblotting. (D) Effect of treatment with the PI3K/MTOR inhibitors alone and in the presence of PD-0332991 on the expression of the indicated proteins was determined by immunoblotting. (E) Quantification of the proliferative effects by CDK4/6 inhibitor concurrent with MEK1/2 inhibitor treatment assessed by Brdu incorporation. Results are expressed as percentage of BrdU Proliferating cells, and a significant difference of p<0.001 was observed between co-treated and MEK1/2 inhibitor treatment alone.

As a whole, PI3K/MTOR inhibitors showed cooperative effects in concert with CDK4/6 inhibitor treatment assessed by viability assay in drug screening. In order to examine the mechanisms, BrdU incorporation was assessed after treatment with BEZ235, GDC0980 and AZD0855 in combination with CDK4/6 inhibitor (Fig 6A). Results indicate that CDK4/6 inhibitor in combination with these PI3K/MTOR inhibitors has a synergistic effect in suppression of cell cycle. PL5 cells that exhibit resistance to both the CDK4/6 inhibitor and PI3K/MTOR inhibitors as single agents displayed a complete proliferative halt after treatment in both acute and long-term responses (Fig. 6B and C). This suppression of proliferation with co-treatment was associated with suppression of Cyclin D1 and Cyclin E1 expression that is observed with PD-0332991 (Fig. 6D). Additionally, there was suppression of additional critical cell cycle regulatory proteins including CDK2 and Cyclin A. In keeping with the suppression of multiple cyclins, although CDK4/6 inhibition has a profound effect on the phosphorylation of RB, there is residual phosphorylation that was further suppressed in the context of co-treatment with PI3K/MTOR inhibitors (Fig. 6E). These data suggest that even low levels of RB phosphorylation can facilitate cell cycle progression, and provide the emphasis for full suppression of CDK-activity through complementary mechanisms.

In addition to PI3K/MTOR, MEK inhibitors also had additive effects in concert with CDK4/6 suppression. We demonstrated the cooperation at the level of cell cycle progression by BrdU incorporation and found that similar suppression of proliferation with the combination treatment (Fig. 6F). The biochemical effect of MEK inhibitors on RB phosphorylation paralleled the observation with PI3K/MTOR inhibition and revealing a similar basis for cooperation (Fig. 6G). Together, these findings suggest that PI3K/MTOR and MEK inhibitors could be particularly in combination with CDK4/6 inhibitors due to complementary mechanisms of action.

## DISCUSSION

The treatment of pancreatic cancer is a particular challenge and new therapeutic approaches are urgently needed. One appealing means of intervention is to target specific genetic features of disease, and CDKN2A loss is one of the most common genetic alterations in PDA. Ostensibly, the loss of the kinase inhibitor results in aberrant CDK4/6 activation that could be targeted through the use of CDK4/6 inhibitors. Here we challenged this simple precept in preclinical models.

Interestingly, and contrary to multiple other tumor models, PDA models exhibited disparate responses to CDK4/6 inhibition that were not dependent on the canonical RB-pathways. In breast cancer, HCC, glioma, melanoma and other models, the response to CDK4/6 inhibition is simply dependent on the presence of RB [31, 33, 51]. Such tumors generally have lost CDKN2A, which provided the rationale for the study herein. Interestingly, within PDA there was a diversity of response to CDK4/6 suppression. Particularly, there were multiple models which exhibited features associated with partial cell cycle inhibition, or relatively complete resistance. These findings are consistent with the work of others [52]. While it would be expected that there would be a full bypass of the requirement for CDK4/6 this was not observed, as RB phosphorylation remained largely dependent on CDK4/6. Additionally, there was significant suppression of a subset of E2F target genes. Interestingly, these genes are largely involved in mitosis, and are controlled by the DREAM and FOXM1 complexes that are ostensibly still effectively repressed in this setting [41]. In fact, we observed similar FOXM1 protein attenuation in all models studied, which is consistent with work demonstrating that FOXM1 protein accumulation is dependent on CDK4/6 activity [40]. However, the control of genes involved in DNA replication has emerged as a key RB-dependent function that is associated with terminal cell cycle arrest [53]. Therefore, the data here suggest that in select tumors models there is an uncoupling of these aspects of cell cycle control. In breast cancer and additional models PD-0332991 results in robust suppression of the full spectrum of E2F target gents and therefore indicates that pancreatic cancer is somewhat unique in this aspect of cell cycle regulatory uncoupling.

The realization that such uncoupling can occur necessitated a highly focused analysis of CDK genes/proteins that drive the G1/S transition. Our group and others have shown that CDK4/6 inhibitors can lead to the accumulation of Cyclin D1 levels [32, 33]. The basis of this response remains under study, but it could reflect more potent mitogenic signaling in the G1-phase of the cell cycle to stimulate the Cyclin D1 promoter. Importantly, we have observed this response in multiple models that exhibit a profound durable response to CDK4/6 inhibition; therefore, Cyclin D1 upregulation does not appear to be associated with resistance to PD-0332991. Surprisingly, our work revealed that resistant models exhibited an aberrant upregulation of Cyclin E. To the best of our knowledge, this is the first time such a response to CDK4/6 inhibition has been observed. In general, CDK4/6 inhibition will reduce the level of Cyclin E transcripts; therefore, the upregulation in resistant models was particularly unexpected. Importantly, we could show that the “levels” of these cyclins contributes to the response and suggests that induction of such proteins will modulate the durable response to CDK4/6 inhibition in a general setting, although Cyclin E-depletion was particularly synergistic with PD-0332991. These results agree with the overall concept that Cyclin E deregulation can contribute to bypass of CDK4/6 inhibitors [54].

In recognition that many PDA models did not harbor a particularly durable response to CDK4/6 inhibition, combination treatments were interrogated. This work revealed that CDK4/6 inhibition has complex interactions on the response to multiple agents. In particular, and consistent with others [55], CDK4/6 inhibition compromised the cytotoxicities of chemotherapies that primarily act via mitotic catastrophe including PLK1 inhibitors and taxanes. This result suggests for this class of drugs it is critical to develop a rational metronomic schedule for drug treatment. In contrast, anti-metabolites are more difficult to predict as RB/E2F regulate a host of genes that are drug targets or otherwise modify dNTP pools [56]. This aspect of RB/E2F function is particularly important when evaluating anti-metabolites that would cooperate or be antagonized by CDK4/6 inhibition. It should be noted that in addition to the enzyme interrogated herein RB/E2F modulates DHFR and RNRII levels that have highly significant effect on dNTP pools.

In contrast with chemotherapy, we observed highly reproducible additive effects across a relatively large panel of MEK and PI3K/MTOR inhibitors with CDK4/6 inhibition. These findings are particularly important since MEK and PI3K/MTOR inhibitors have been considered for the treatment of pancreatic cancer [57]. As shown here CDK4/6 inhibition can have relatively subtle effects on cell cycle and suppression of proliferation. In contrast, drug combinations yielded potent cytostatic response in all models tested. In the case of PI3K/MTOR the induction of Cyclin D1 and Cyclin E that occurs with CDK4/6 inhibition was completely blocked resulting in profound de-phosphorylation of RB. These finding are consistent with the work of others showing that cyclin D1 levels are under the control of MEK or MTOR signaling [58, 59]. Presumably, these data suggest that one of the “weaknesses” of pharmacological CDK4/6 inhibitors is compensatory or alternative regulators of RB phosphorylation. In the case of MEK and PI3K/MTOR, there are well described mechanisms through which they limit the expression of Cyclins and suppress CDK activity. Ostensibly in many therapeutic contexts, these mechanisms of attenuating CDK-activity are NOT sufficient to halt cell cycle; however, due to the complementary mechanism of action such agents are particularly effective in concert with CDK4/6 inhibitors. Likely, a similar mechanism could underlie the profound clinical activity of CDK4/6 inhibitors with endocrine therapy; since in ER-positive breast cancer estrogen signaling is required for the expression of cyclin D1 and E [60, 61]. Together, the work herein provides a roadmap for considering the clinical utilization of CDK4/6 inhibitors in the treatment of PDA.

## MATERIALS AND METHODS

### Cell culture, chemicals and antibodies

Human pancreatic cancer cell lines PL45, MIAPACA-2, PANC1, CAPAN2, BXPC3, HS 766T, ASPC1, PL5 were purchased from the American Type Culture Collection (ATCC). All cells except for MIAPACA2 were cultured in DMEM supplemented with 10% fetal bovine serum and antibiotics. MIAPACA2 cells were grown in DMEM with fetal bovine serum and horse serum to a final concentration of 10% and 2.5% respectively plus antibiotics. Drugs used in this study were purchased from Selleck Chemicals. Compounds were diluted in DMSO to 5 mM stock concentrations. PD-0332991 was employed at a concentration range of 0.5-2 μM. Drugs screen was performed at 2.5 μM. Antibodies against CDK2, CCNE1, CCNA2, GAPDH, total ERK, p-ERK, lamin B, DCK, TS and p27 were purchased from Santa Cruz biotechnology. While, antibodies against total RB, p-RB(780), total Akt, p-Akt (ser473) were purchased from Cell signaling. Cyclin D1 (AB3) was from NeoMarkers.

### Drug Screen

A library of 304 drugs from Selleck Chemicals was used for drug screening. Cells were grown in 96-well plates at 2500 cell per well. Subsequent to plating, cell were treated with 1μM PD-0332991 or DMSO for 24 hours at 37° and 5.0 CO_2_. The next day, cells were treated with the drug library to final concentration of 2.5 uM, and incubated at 37° and 5.0 CO_2_ for 72 hours. Following treatment, viability was assessed using Celltiter Glo(Promega). The relative fluorescence units were detected and analyzed by a BIOTEK plate reader and Gen5 software. Data analysis was performed using Drug-decode macro (developed by David Haan, UT Southwestern).

### Flow cytometry

Cell were trypsinized and fixed in 70% Ethanol post a 2 hour incubation with Brdu labeling solution(Invitrogen). Fixed cells were incubated in 2M HCl with 0.3mg/ml pepsin solution for 30 minutes followed by addition of 0.1 M sodium tetraborate solution(pH 8.5). After acid neutralization, cells were washed twice with IFA(10mM HEPES(pH 7.4), 25 mM NACI, 4% FBS) buffer. Cells were then incubated with FITC labeled anti-Brdu(BD Pharmingen) at 1:10 dilution for 30 min at room temperature. After Brdu labeling, Propidium iodine(20ug/ml) and RNAse(4ug/ml) was added and incubated for 15 minutes in Dark. BrdU incorporation was assessed using a Facscalibur Instrument and data was analyzed using FlowJo.

### Western blot analysis and reverse phase protein array analysis

Pancreatic cell lines were lysed in RIPA buffer(10% NP-40 substitute, 1% SDS, 500mM Tris-HCI(pH 7.4), 1.5M NaCI, 5% sodium doxycholate, 10mM EDTA) containing both complete protease inhibitor cocktail tablets (Roche) and phosphatases inhibitors (Roche). Protein concentrations were subsequently measured with the BCA protein assay DC^tm^ Protein Assay(BIO-RAD) using manufacture's protocol. These samples were separated on SDS polyacrylamide gels and electroblotted on to Immobilon-P Transfer Membrane. Membranes were washed with wash buffer (0.1% Tween-20/1x saline). Followed by incubation with blocking solution (5% BSA, 0.1% Tween-20; saline), followed by incubation with rabbit primary antibodies (1/500 dilution) at 4°C overnight. The membranes were then washed in Wash buffer and incubated with horseradish peroxidase conjugated secondary antibodies (Jackson ImmunoResearch) for one hour at room temperature. Antibody detection was performed with an enhanced chemiluminescence reaction (SuperSignal) as directed by manufacture.

Protein lysates were prepared from cell treated with vehicle or 1 μM PD-0332991 for 48 hours. Preparation of the lysates were as directed by the MD Anderson RPPA shared service, and the analysis of the signals were performed using their highly standardized procedures. The difference in normalized log protein values are presented in the analysis.

### Short-term and long-term treatments of in vitro models

For short term treatments, pancreatic cell lines were seeded at 5×10^4^ cell/well in 6 well plate and left to adhere for 3 hours at 37 °C. Cells were then treated with individual drug or drug combinations at 1uM concentrations and incubated for 24-72 hours. For long-term experiment, cell were seeded at 5×10^3^ cell/ well and drugs were replenished every 3 days for 10 days. Following long term studies, they were assessed by cystal violet.

### Transfection of siRNA

Pancreatic cell lines were seeded in a 6-well plate at a desity of 5×10^5^ cells per well and transfected with siRNA against either CCND1 (Santa Cruz biotechnology), CCNE1 (SMARTpool: Accell CCNE1 siRNA; Dharmacon) and PLK1(SMARTpool: Accell PLK1 siRNA; Dharmacon) at a final concentration 50nmol/L with Lipofectamine RNAiMAX(Invitrogen) according to manufacture's instructions. Transfections with a control sRNA(TMEM) served as a negative control.

### Gene Expression analysis

Cells were treated with vehicle or 1μM PD-0332991 for 48 hours (RBTARGET) or 120 hours (Agilent Array). The RBTARGET array is a custom Affymetrix Gene Chip that encompasses genes regulated by RB and highly correlated genes together with additional gene for normalization, full list of the probes on the RBTARGET platform are provided in the supplemental data. Total RNA was recovered and hybridized using standard procedures. Data analysis was performed on normalized data. To define differentially expressed genes, a cutoff of 2-fold and p<0.05 was utilized.
